# PD-1 monoclonal antibodies as an effective treatment for rare cutaneous malignancies: a case report

**DOI:** 10.3389/fonc.2025.1609767

**Published:** 2025-06-24

**Authors:** Ryan Falk, Wolfram Samlowski, Amin Hedayat

**Affiliations:** ^1^ Kirk Kerkorian School of Medicine, University of Nevada Las Vegas (UNLV), Las Vegas, NV, United States; ^2^ Nevada Oncology Specialists, Las Vegas, NV, United States; ^3^ Department of Internal Medicine, University of Nevada School of Medicine, Reno, NV, United States; ^4^ Quest Diagnostics, Associated Pathologists Chartered, Las Vegas, NV, United States; ^5^ American Melanoma Institute, Las Vegas, NV, United States

**Keywords:** checkpoint inhibitor, cemiplimab, pembrolizumab, adenosquamous carcinoma, sebaceous carcinoma, adnexal carcinoma

## Abstract

Adnexal carcinoma and adenosquamous carcinoma are rare forms of skin malignancy that are usually treated via surgical resection or radiotherapy. These cancers become clinically challenging when localized treatment is not feasible. In addition, the cosmetic and functional defects resulting from radical surgical resection of tumors in peri-orbital areas need to be considered. The checkpoint inhibitors pembrolizumab and cemiplimab have been effective treatments for a number of cutaneous malignancies. We present cases of adenosquamous skin cancer and eyelid sebaceous gland cancer that achieved rapid, complete pathological and radiological remission in response to treatment with single agent PD-1 monoclonal antibodies. These patients had minimal, if any toxicity associated with treatment and have each remained recurrence-free for over a year of follow up. The therapeutic potential of PD-1 monoclonal antibodies as a treatment for these rare skin malignancies warrants further evaluation in clinical trials.

## 1 Introduction

Monoclonal antibodies directed against the inhibitory immune checkpoints PD-1, PDL-1 and CLTA-4 have demonstrated substantial activity in the treatment of a variety of skin malignancies. For example, durable responses and prolongation of survival have been observed in patients with advanced melanoma following PD-1 antibody monotherapy with pembrolizumab and nivolumab (1, 2). Combination therapy of metastatic melanoma with nivolumab plus the CLTA-4 antibody ipilimumab has further increased melanoma-specific survival to >50% with 10-year follow-up (3). More recently, PD-1 antibodies cemiplimab and pembrolizumab have induced significant clinical responses and progression-free survival in locally advanced or metastatic keratinocyte-derived carcinomas (squamous and basal cell carcinomas) (4–6). A high tumor response rate has also been demonstrated in metastatic Merkel cell carcinoma, following avelumab (a PDL-1-directed antibody) or pembrolizumab treatment (7, 8). These reports have demonstrated significant clinical activity of immune checkpoint inhibitor (ICI) treatment in these more common types of skin cancer. Unfortunately, little is yet known about the usefulness of ICI therapy in rarer skin malignancies.

We report a case of metastatic adenosquamous carcinoma and a case of locally advanced adnexal carcinoma. Both patients achieved a radiologic complete remission following treatment with pembrolizumab monotherapy. Based on our experience, further evaluation of ICI in these uncommon skin cancer malignancies appears warranted.

## 2 Case reports

### 2.1 Case 1

The patient was an 86 year old man with a history of arthritis, gout, back pain, hypertension, coronary artery disease, as well as renal, liver and lung problems. This patient had a history of many recurrent basal and squamous cell carcinomas, as well as a prior superficial melanoma.

In 2020, the patient developed a large infiltrative squamous cell carcinoma of the right chest with regional lymph node metastases. He was treated into remission with 7 doses of cemiplimab. Consolidative RT was also administered to the chest lesion. Treatment was electively discontinued 9/29/21 after a radiologically confirmed remission. This lesion never recurred.

**Figure 1:**
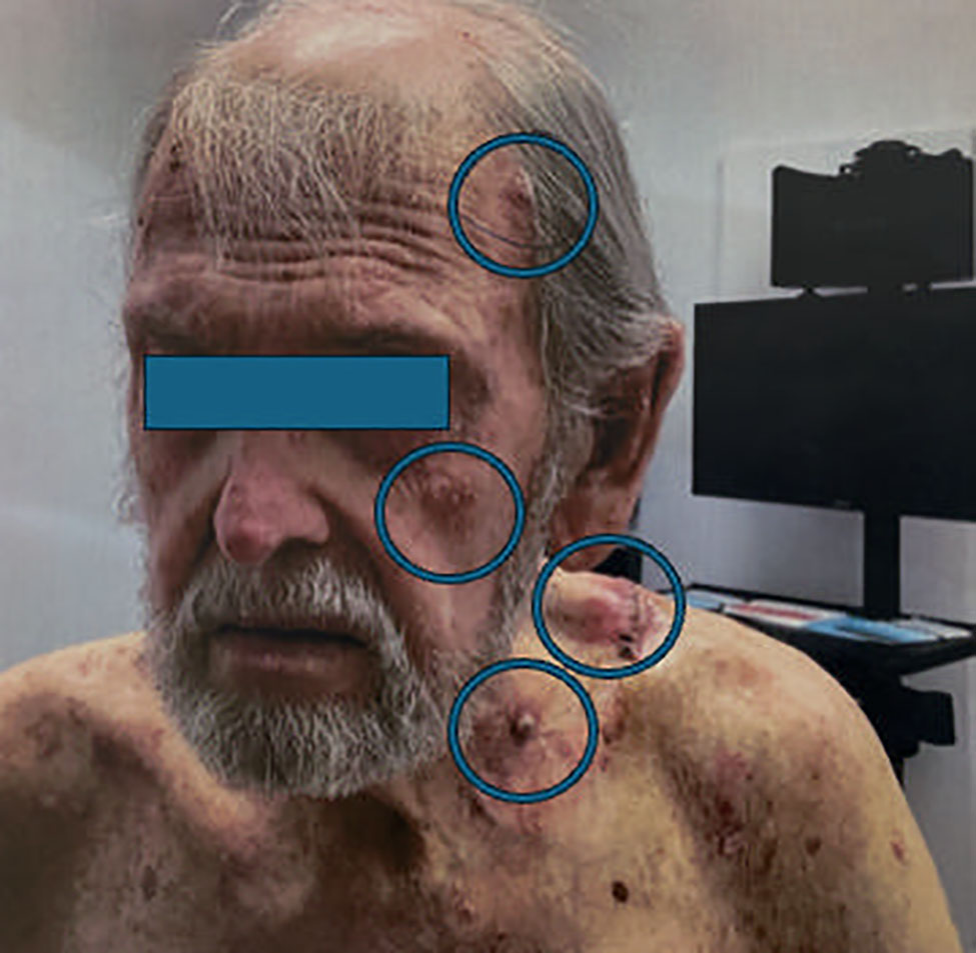
Clinical photograph demonstrating numerous subcutaneous metastases from adenosquamous skin cancer (encircled).

**Figure 2:**
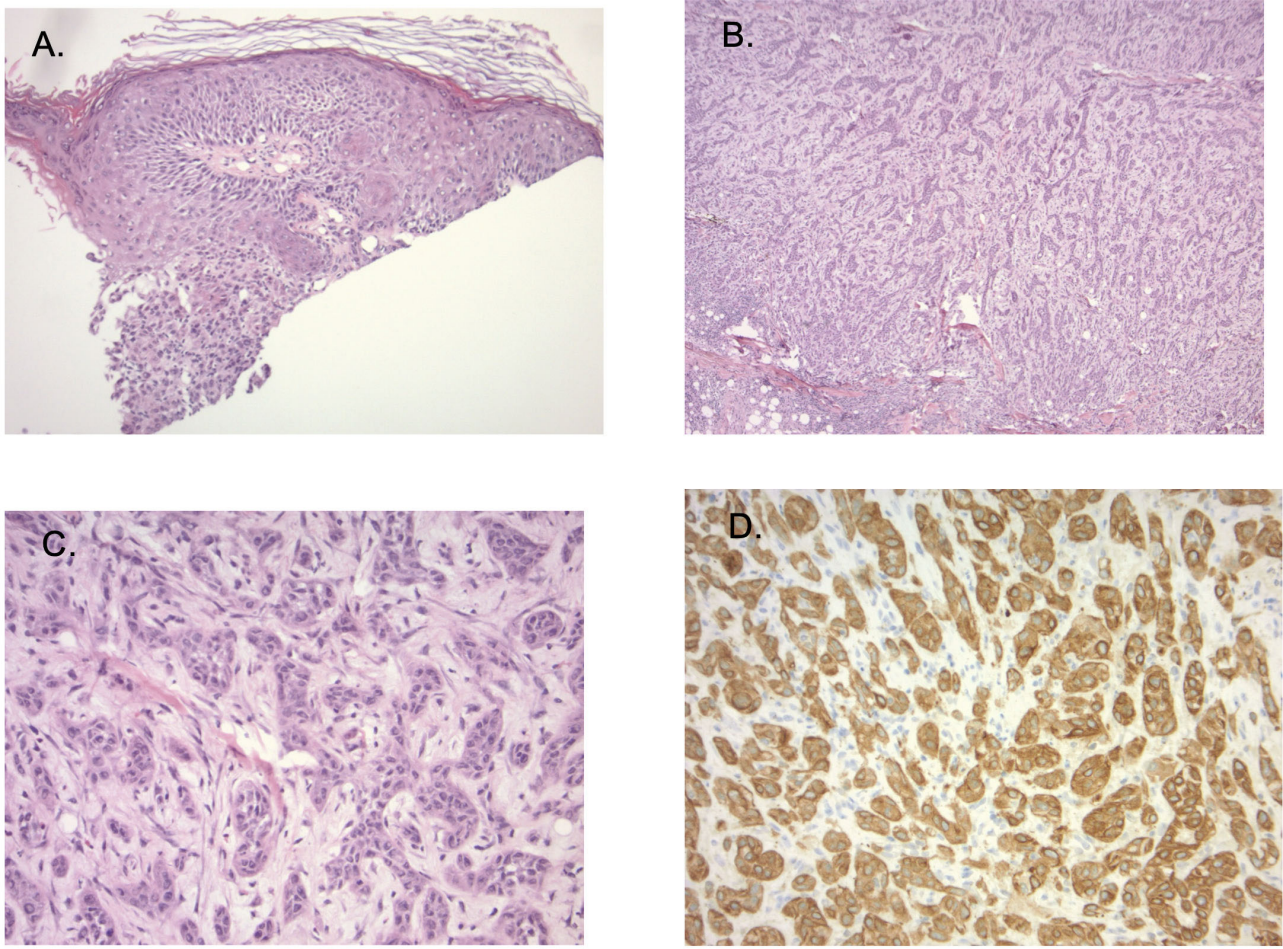
**(A)** Sections show ulcerated skin (40X magnification). **(B)** tumor consists of dermal cords and nests of atypical epithelioid cells that present in sclerotic (100X magnification), **(C)** (200X magnification) showing hyalinized stroma, and areas with lumen-like differentiation. Secondary inflammatory changes are prominent. **(D)** (200X magnification): Immunostaining showing extensive CK5/6 expression, while S100, Ber-EP4, EMA and CK7 staining was negative in lesional cells.

**Figure 3:**
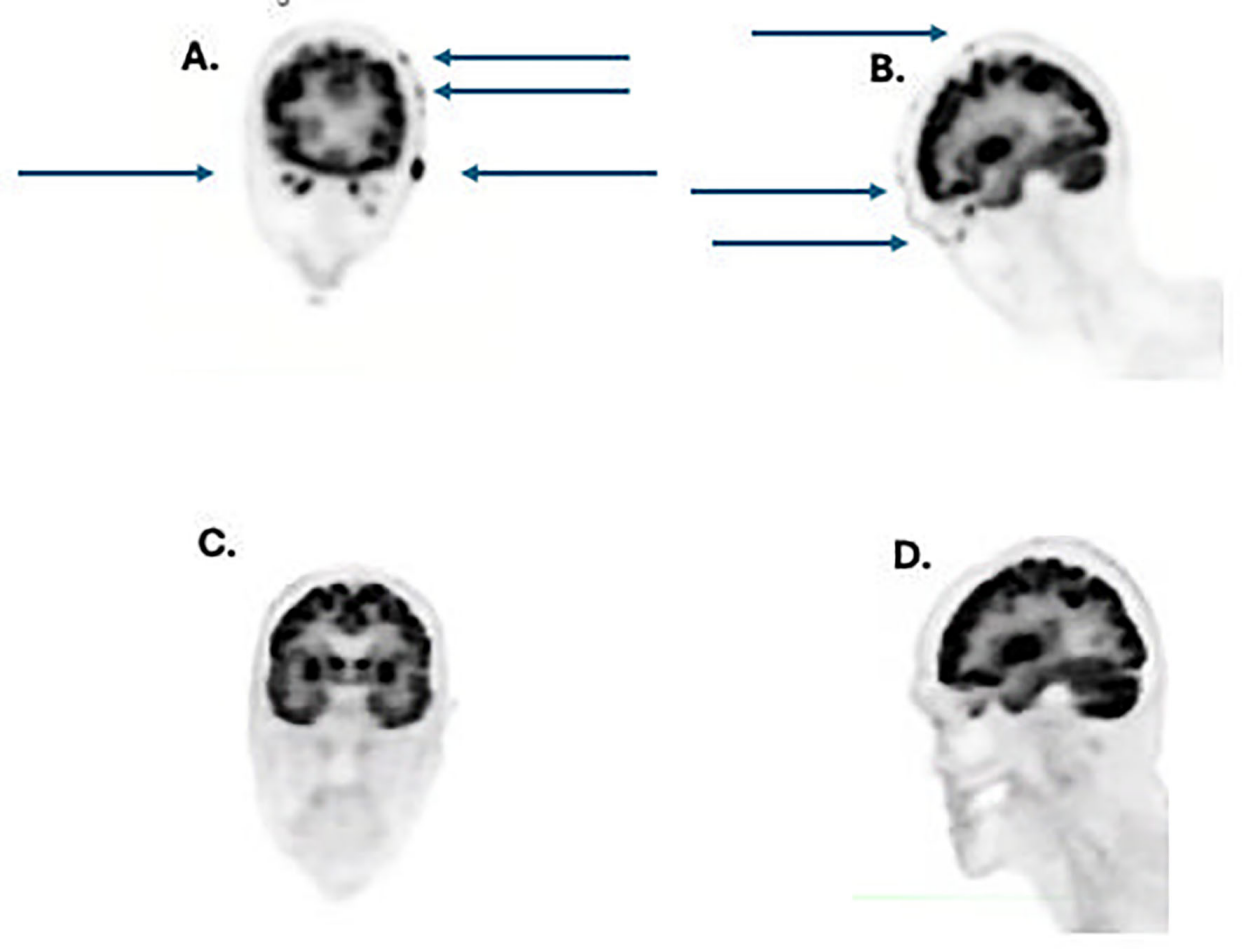
**(A)** coronal view pretreatment showing numerous tumor metastases (arrows); **(B)** lateral view pretreatment showing numerous tumor metastases (arrows); **(C)** coronal view after 4 doses of cemiplimab showing complete resolution of tumor metastases, **(D)**, lateral view post-treatment showing complete regression of tumor metastases.

In 03/02/2023: The patient developed numerous growing painful and bleeding lesions on the scalp, neck and shoulder (**Figure 1**). A biopsy of a scalp lesion demonstrated metastatic adenosquamous skin carcinoma (**Figure 2**). Initial PET/CT imaging is shown (**Figures 3A, B**). Treatment with oral capecitabine (1000 mg/m2, days 1-14) was initiated. The skin lesions failed to respond. The patient was admitted to the hospital due to toxicity from capecitabine treatment including fevers, chills, nausea, vomiting, profound diarrhea, and neutropenia.

Due to the prior successful use of cemiplimab with minimal toxicity, cemiplimab was restarted 4/14/23. After 3 doses (350 mg every 3 weeks i.v.), the patient achieved a dramatic clinical and radiographic complete response of his scalp lesions, as well as and neck and shoulder lesions. A PET scan from 8/2023 is shown (**Figures 3C, D**). The remission of this cancer proved durable for 7 additional months following initial identification of a complete remission on PET/CT scan without recurrence of this neoplasm.

The patient’s course was subsequently complicated by development of yet another skin malignancy. He developed an undifferentiated pleomorphic sarcoma of the left upper arm skin. This tumor underwent a wide excision but progressed with distant metastases. The patient declined sarcoma-directed chemotherapy. Unfortunately, this tumor failed to respond to ongoing cemiplimab or pazopanib treatment. The patient eventually died on March 8, 2024, due to progressive metastatic sarcoma without recurrence of his prior adenosquamous carcinoma.

### 2.2 Case 2

The patient was a 66-year-old woman with a history of pre-diabetes, hypertension, Hashimoto’s thyroiditis, post-concussion syndrome sustained after a head injury, with residual right trigeminal neuralgia.

The patient developed a nodule in her left eyelid in 2019 that was treated conservatively with localized therapy and massage. This lesion gradually progressed to involve a substantial part of the left upper eyelid, including the region of the medial canthus and tear-duct.

**Figure 4:**
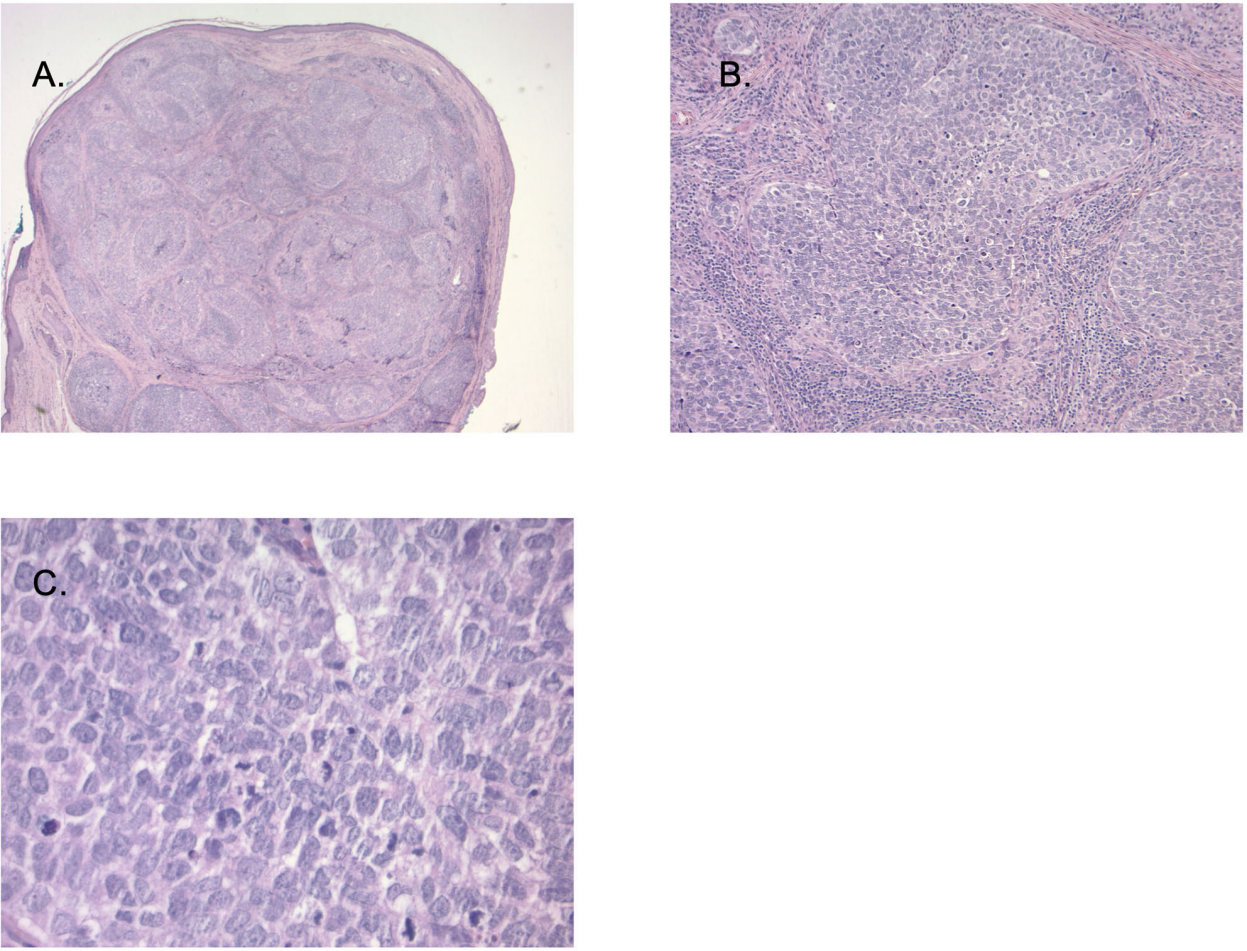
**(A)** (20X magnification): Sections demonstrate a nodular tumor occupying a Meibomian gland with intra conjunctival carcinoma. Image **(B, C)** (100X magnification and 400X magnification) Tumor cells are large with high grade atypia including high nuclear: cytoplasmic ratio, pleomorphism, hyperchromasia, and numerous mitotic figures. Immunohistochemical stains (not shown) demonstrated strong cytokeratin 7 and variable diffuse androgen receptor (AR) expression (not shown).

**Figure 5:**
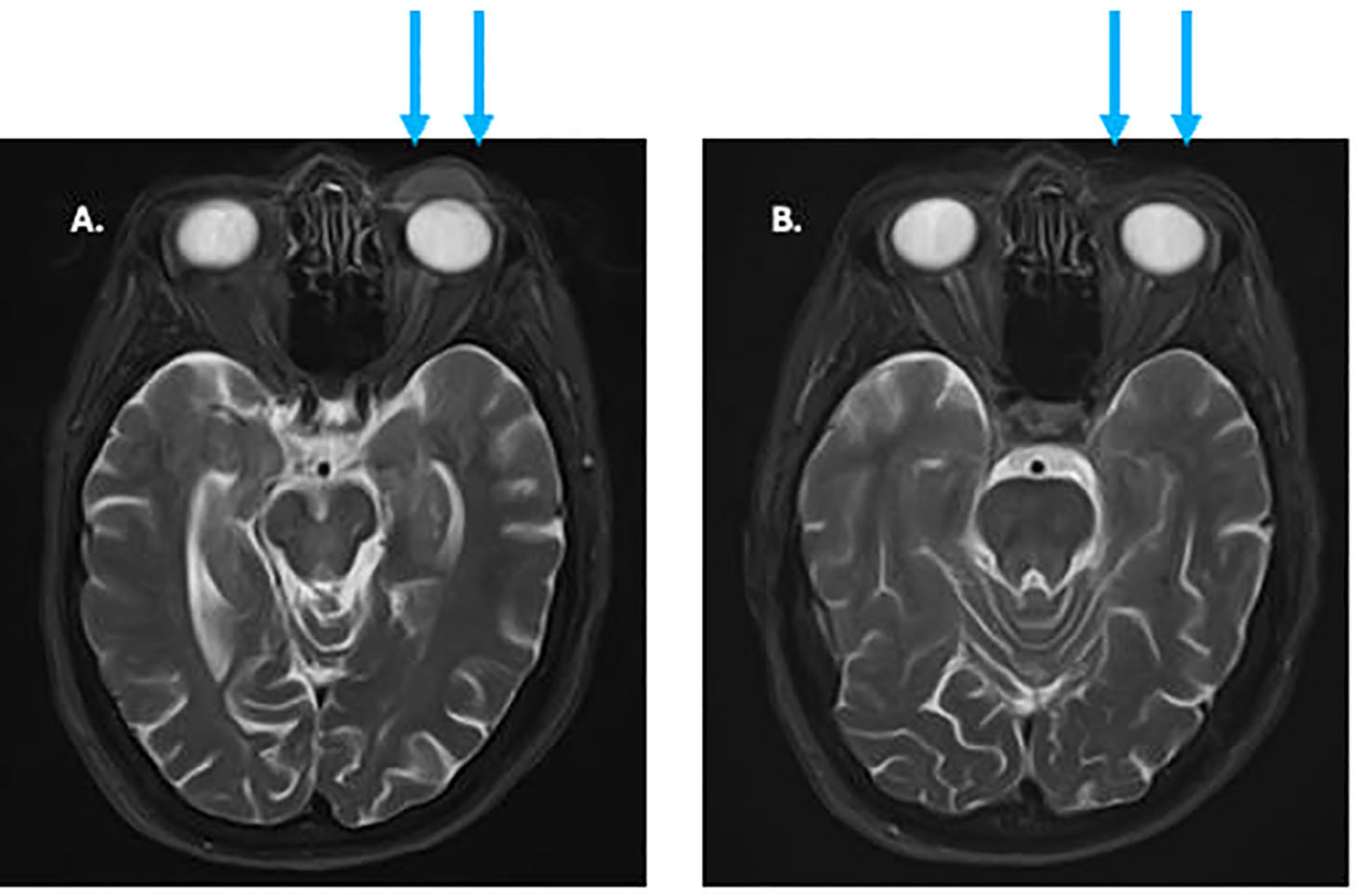
**(A)** pre-treatment MRI of facial soft tissues showing eyelid tumor (arrows). **(B)** MRI after 3 months of treatment showing complete regression of previous tumor (arrows).

A biopsy was performed identifying a poorly differentiated carcinoma that was felt to be a sebaceous carcinoma (**Figure 4**). In 2023 PET and MRI imaging were performed to define the extent of the mass (**Figure 5A**). Molecular testing (Foundation Medicine, showed a PDL-1 tumor proportion score of 0%, a TMB of 2/Mb and no targetable oncogene mutations. The patient was treated with neoadjuvant pembrolizumab, with the intent of down-sizing the tumor to decrease the scope of surgery necessary in this anatomically sensitive location. After 4 doses of pembrolizumab, the patient had complete regression of her tumor. This was confirmed by MRI scan after the 4^th^ dose of pembrolizumab (**Figure 5B**). Planned surgical resection of the tumor site was omitted, due to apparent radiologic complete remission. The patient received 5 additional doses of pembrolizumab as consolidation therapy post-remission. After a confirmatory negative MRI scan, she underwent elective treatment discontinuation, based on our institutional protocol (9). She currently remains disease-free after over 18 months of additional follow-up.

Immunohistochemical staining of tissue sections was performed using the following primary antibodies from Dako (Agilent Technologies): CK5/6 (clone D5/16 B4, M7237), S100 (polyclonal, Z0311), Ber-EP4 (clone Ber-EP4, IS637), EMA (clone E29, M0613), CK7 (clone OV-TL 12/30, M7018), and AR (clone AR441, M3562). Detection was carried out using the EnVision™+ system with appropriate positive and negative controls.

## 3 Discussion

Previous reports have suggested potential clinical benefit from ICI treatment of some rare cutaneous malignancies (10, 11). For example, ICI treatment has become a standard of care for treatment of advanced Merkel Cell Carcinoma (12).

The use of ICI in the treatment of additional rare cutaneous malignancies remains an area of current investigation. Adenosquamous carcinoma (ASC) is a highly aggressive variant of squamous cell carcinoma (SCC), characterized by local invasiveness (13). Unlike more common cutaneous SCC, adenosquamous cancers most frequently arise in organs, such as the uterus and lungs. Skin primaries in the head and neck region are rare (14). Standard treatment options for localized cutaneous ASC include surgical resection or radiotherapy (RT), with reported three-year progression-free survival (PFS) rates of 45.6% for primary surgery and 83.3% for definitive RT for localized lesions (15). In contrast, there appears to be minimal published experience with systemic therapy for metastatic adenosquamous carcinomas (13).

Similarly, adnexal carcinoma is a rare, locally aggressive malignancy which is believed to originate from sweat glands, in tissues such as the eyelids (16). Mohs micrographic surgery (MMS) is considered the standard treatment for localized disease; however, survival and recurrence data for adnexal carcinomas, particularly those affecting the eyelid, remain limited (17, 18). In cases where MMS is not a viable option, RT may be considered, but the radioresistant nature of these tumors often leads to suboptimal outcomes (16). Consequently, patients with unresectable tumors, particularly in sensitive anatomical locations, such as the eyelid, face significant therapeutic limitations.

Exploration of checkpoint inhibitor therapy of rare cutaneous malignancies is ongoing (10). There is one prior report of successful ICI treatment of a patient with metastatic adnexal carcinoma with high PDL-1 expression (19). Our eccrine carcinoma patient had a PDL-1 tumor score of 0% and a low tumor mutation burden, which are generally felt to be adverse markers for ICI treatment response. There is another report of a lengthy response of metastatic sebaceous carcinoma following pembrolizumab treatment (20). There is also precedent for responses of porocarcinoma, another rare skin malignancy, with ICI therapy (21, 22).

Our two cases demonstrate the potential efficacy of ICI in achieving complete remission in rare skin cancer variants, despite challenging clinical presentations. In Case 1, treatment of metastatic adenosquamous carcinoma with chemotherapy was initially attempted; however, the patient’s age and frailty resulted in severe toxicity and hospitalization, precluding further chemotherapy treatment. In case 2, the patient would have required a large cosmetically unfavorable surgical resection, due to encroachment of the tumor on the lacrimal duct and involvement of the entire upper eyelid. Treatment with pembrolizumab in both patients achieved durable complete remissions.

In conclusion, our case report highlights the potential activity of ICI for treatment of rare histological types of skin cancer. We frequently treat patients with 1–2 cycles of neoadjuvant treatment (approximately 6 weeks), regardless of PDL-1 and tumor mutation burden status, to assess tumor response. It seems reasonable to continue to treat responding patients with ICI, as they potentially can achieve a durable complete remission. In non-responding patients we proceed to definitive surgical resection or radiotherapy. Our current case report should be considered hypothesis generating due to the limited number of patients. While neither of our cases experienced ICI-related toxicity, it is also important to emphasize that the potential risk of immunologic toxicity requires close patient monitoring.

## Data Availability

The raw data supporting the conclusions of this article will be made available by the authors, without undue reservation.
